# Cytokine-, Neurotrophin-, and Motor Rehabilitation-Induced Plasticity in Parkinson's Disease

**DOI:** 10.1155/2020/8814028

**Published:** 2020-11-26

**Authors:** Gabriella Policastro, Matteo Brunelli, Michele Tinazzi, Cristiano Chiamulera, Dwaine F. Emerich, Giovanna Paolone

**Affiliations:** ^1^Department of Diagnostic and Public Health, University of Verona, Verona, Italy; ^2^Department of Neuroscience, Biomedicine and Movement, University of Verona, Verona, Italy; ^3^Glocester, Rhode Island, USA

## Abstract

Neuroinflammation and cytokine-dependent neurotoxicity appear to be major contributors to the neuropathology in Parkinson's disease (PD). While pharmacological advancements have been a mainstay in the treatment of PD for decades, it is becoming increasingly clear that nonpharmacological approaches including traditional and nontraditional forms of exercise and physical rehabilitation can be critical adjunctive or even primary treatment avenues. Here, we provide an overview of preclinical and clinical research detailing the biological role of proinflammatory molecules in PD and how motor rehabilitation can be used to therapeutically modulate neuroinflammation, restore neural plasticity, and improve motor function in PD.

## 1. Introduction

PD is the second most common neurodegenerative disorder generally affecting the population over 65. In fact, only 4% of cases occur before the age of 50 [1]. The disease is induced by the loss of nigrostriatal dopaminergic neurons, intracellular *α*-synuclein accumulation, and onset of motor symptoms such as abnormal voluntary movements, tremor, rigidity, slowness of movement, postural instability [2], and nonmotor impairments including cognitive decline [3], depression, and sleep disturbances [4].

However, recently, postmortem brain imaging and fluid biomarker investigations identified neuroinflammation as a crucial pathogenesis factor of PD [5–7]. Neuroinflammation is marked by activated microglia and reactive astrocytes within brain parenchyma and by the release of various inflammatory mediators including cytokines, chemokines, reactive oxygen species (ROS), and reactive nitrogen species (RNS) [8]. These mediators can be secreted by microglia in the central nervous system (CNS), peripheral immune cells, and other cell types such as dysfunctional adipocytes [9, 10], sustaining the inflammatory reaction and maintaining a self-reverberating cycle. For a long time, the blood-brain barrier (BBB) was thought to be unaffected by neurodegenerative and neurological pathologies while nowadays, a growing body of evidence suggests that the BBB is pathologically modulated, allowing the penetration of peripheral macrophages, leukocytes, and systemic proinflammatory mediators, such as monocyte chemotactic protein-1 (MCP-1), tumor necrosis factor-*α* (TNF-*α*), interleukin-1*β* (IL-1*β*), interleukin-8 (IL-8), and interferon-*γ* (IFN-*γ*) [11–15]. The overproduction of proinflammatory mediators also reduces the production of brain plasticity-related molecules, such as brain-derived neurotrophic factor (BDNF) and glial cell line-derived neurotrophic factor (GDNF), and the ability of CNS to adapt in response to a variety of external stimuli [16, 17]. In addition, in recent years, researchers have focused their attention on the beneficial effect of physical exercise on PD patients suggesting that exercise, through targeted training, can increase neuroplasticity and, in turn, improve patients' motor and cognitive performance [18]. Here, we intend to explore (1) the role of proinflammatory cytokines and (2) the impact of traditional and not traditional forms of physical exercise on neuroinflammation and neuroplasticity in parkinsonian subjects undergoing motor rehabilitation.

To reach the aim of this study, publication search for literature review was conducted using the NCBI PubMed database based on the following groups of keywords: (1) Parkinson's disease, pro-inflammatory cytokines; (2) Parkinson's disease, IL-6, IL-1*β*, IL-8, MCP-1, and TNF-*α*; (3) Parkinson's disease, neuroinflammation, neuroplasticity; (4) Parkinson's disease, physical activity, neurorestoration, neuroplasticity; (5) Parkinson's disease, exercise, neurotrophic factors; (6) Parkinson's disease, exercise, BDNF; (7) Parkinson's disease, exercise, GDNF; (8) Pro-inflammatory cytokines, exercise, PD patients; and (9) Not traditional physical exercises, inflammatory state, PD patients. To be eligible for inclusion in the review, studies must have been published between 1990 and 2020.

## 2. Proinflammatory Cytokines in PD

Brain cytokine activity depends on several conditions such as the cellular sources and the pathophysiological context all contributing to the effects exerted on the brain. In fact, cytokines can promote apoptosis of neurons, oligodendrocytes, and astrocytes; cause damage to myelinated axons; but even initiate neuroprotective effects, independently of their immunoregulatory properties [19]. Although to date there is no evidence to support a specific role for any particular cytokine as a direct cause of neurodegenerative conditions, cytokine-driven neuroinflammation and neurotoxicity have been shown to modify the disease progression.

Among cytokines, interleukin-6 (IL-6), IL-1*β*, IL-8, MCP-1, and TNF-*α* have been the most studied in PD.

### 2.1. IL-1*β*

IL-1*β* is a proinflammatory cytokine produced mainly by macrophages and monocytes [20] and also by epithelial cells [21] and endothelial cells [22], and it has a key role in regulating inflammatory response to microbial stimuli such as the lipopolysaccharide (LPS) and sterile insults (e.g., hypoxia, hyperosmolarity, thermal damage, and gamma radiation) [23, 24].

It has been demonstrated that IL-1*β*, a part of the IL-1 family, acts on the CNS because of the permeability of the BBB [25], and it is also secreted into the CNS by microglial cells [26–28], astrocytes [29], oligodendrocytes [30], and neurons [31, 32]. Therefore, the presence of members of the IL-1 family and in particular IL-1*β* and its receptor in basal conditions in the CNS could suggest a normal physiological role for IL-1*β*. For instance, several studies demonstrate that IL-1*β* stimulates astrocytes and supports neuronal survival via production of neurotrophic factors [33, 34]. However, IL-1*β* contributes to and/or sustains the pathological processes and results upregulated in several neurodegenerative diseases. Increased IL-1*β* levels have been detected in the cerebrospinal fluid (CSF) and the striatum of postmortem PD patients [35] as compared to control subjects. Moreover, studies based on adenoviral vectors reported that sustained expression of IL-1*β* in the substantia nigra (SN) causes irreversible and pronounced dopaminergic neuronal loss and motor symptoms [36, 37], while IL-1*β* increase induced by acute administration of LPS in the SN was not toxic [38, 39]. Overall, these data suggest that sustained but not acute IL-1*β* expression has toxic effects on the SN. In addition, loss of tyrosine hydroxylase- (TH-) positive neurons was higher in animals that received both, a stimulus of LPS in the SN and 6-OHDA injection into the striatum, compared to those receiving just an acute stimulus of LPS [39].

Nonetheless, Saura and colleagues have demonstrated that an acute infusion of a high dose (20 ng) of IL-1*β* in the SN of rats 5 days before the injection of 6-hydroxy dopamine (6-OHDA) in the striatal region protects dopaminergic cellular bodies from 6-OHDA, does not induce microglia activation, and prevents motor dysfunctions [40]. Therefore, although most of the evidence reveals that an inflammatory stimulus previous to a neurodegenerative treatment increased neuronal cell death [36, 37, 39], under specific circumstances, protective effects cannot be ruled out.

### 2.2. IL-6

IL-6 is a member of the neuropoietic cytokine family with a wide range of biological activities. It is involved in the development, differentiation, degeneration, and regeneration of neurons in the central and peripheral nervous systems and can also stimulate glial cells [41, 42]. Dysregulation of IL-6 production and signalling has also been reported in several neurodegenerative diseases, including PD [43–45]. Interestingly, IL-6-mediated neuronal degeneration in the CNS [46] and IL-6-mediated biological activities [47, 48] depend, respectively, on the activation of two different types of IL-6 pathways: the “ transsignalling “and “classical signalling.” Classical signalling occurs when the 80 kD subunit of the IL-6 receptor, called IL-6r, binds to the protein. The binding of IL-6 to IL-6r is followed by homodimerization of the second receptor subunit, called gp130, and by the activation of two distinct signalling pathways: (1) the Janus kinase- (JAK-) signal transducer and activator of transcription (STAT) pathway (JAK/STAT signalling pathway) and (2) the mitogen-activated protein kinase (MAPK)/extracellular signal-regulated kinase (ERK) signalling pathway [49–51]. While IL-6r is only expressed by hepatocytes, neutrophils, monocytes/macrophages, and specific lymphocyte subpopulations [52], IL-6 affects many more cell types. This is possible because of “transsignalling”: IL-6r exists in a soluble form, sIL-6r, which can bind to IL-6 and develop a circulating IL-6/sIL-6r complex which can induce the dimerization of the gp130 even in cells that do not possess IL-6r. Activation of the IL-6 pathway by IL-6/sIL-6r is known as transsignalling [53, 54]. The two pathways lead to two different cellular responses [55]. The classical pathway mediates anti-inflammatory signals while the transsignalling pathway mediates proinflammatory signals (e.g., IL-6 mediates neurodegeneration [46], cancer inflammatory response in the colon [56], and inflammatory bowel disease [57]). This emphasizes the importance of distinguishing between the two pathways when prescribing drugs for the treatment of neurological or neurodegenerative diseases [58].

In regard to the expression of IL-6 in PD, there are some controversial results. Several studies observed an increase of IL-6 in the nigrostriatal region of the postmortem brain and in CSF of PD patients [35, 43, 59, 60]. In some studies, no difference in plasmatic levels of IL-6 was reported [60, 61], while others found elevated levels in PD patients with severe depression [62]. Still, one paper reported that IL-6 was at higher plasmatic levels in patients with a rapidly progressing disease compared to patients with usual progression [62]. Interestingly, it has been shown that Levodopa, in physiological concentrations, elicits an immunomodulatory effect on cells from both PD patients and controls and caused stimulation of IL-6 production [44].

### 2.3. IL-8

IL-8 is a chemoattractant cytokine secreted by a variety of cells (e.g., monocytes [63], macrophages [64], endothelial cells [65], dermal fibroblasts [66], keratinocytes [67], hepatoma cells [68], synovial cells [69], and chondrocytes [70]), and it is well known as an inflammatory factor which induces a chemotactic response involving infiltration of neutrophils through the BBB [71]. Moreover, activated microglia is also a potent secretory source of IL-8 and expresses CXCR2 receptor for the chemokine providing a positive feedback mechanism for sustained amplification of inflammatory response [72]. At present, few studies have examined levels of IL-8 in the PD brain. In one study of Koziorowski and collaborators, serum levels of the chemokine were measured in individuals diagnosed with idiopathic PD and in controls. The results showed that IL-8 concentrations were doubled in the diseased brain compared with the control; this difference in levels of the chemokine was significant [73]. However, a contrary finding has recently been reported. Levels of IL-8 and cytokine TNF-*α* were found reduced in serum from Indian PD patients relative to controls [74]. Overall, despite the relevance of neuroinflammation in the pathophysiology of PD, data is lacking on the roles of IL-8 and other chemotactic factors in the progression of the disease.

### 2.4. MCP-1 (CCL2)

MCP-1 (CCL2), one of the most highly and transiently expressed chemokines during inflammation, is a member of the CC subtype chemokines. MCP-1 exerts its biological functions by binding to its high-affinity receptor, CCR2, which is mainly expressed by microglia, astrocytes, and brain microvascular endothelial cells (BMECs) in the brain [75, 76].

Several studies have demonstrated that MCP-1 is constitutively present in the brain. The neuronal expression of MCP-1 is mainly found in the cerebral cortex, globus pallidus, hippocampus, lateral hypothalamus, Purkinje cells, cerebellum, astrocytes, perivascular microglia, infiltrating leukocytes, cholinergic neurons of magnocellular preoptic, and in dopaminergic neurons of the substantia nigra pars compacta [77, 78]. The low expression in discrete neuroanatomical regions with classical neurotransmitters or neuropeptides suggests that MCP-1 may act as a modulator of neuronal activity and neuroendocrine functions [79].

Additionally, MCP-1 may modulate the function of the BBB components and thus affect the integrity of BBB. In accordance with this hypothesis, the MCP-1 level has been found to positively correlate with the permeability of the BBB and progression of diseases [80, 81] while the lack of MCP-1 or CCR2 prevents neuronal death, decreases BBB permeability, and improves neuronal function in some disorders, including hemorrhage and ischemia-reperfusion injury [81, 82].

It has also been established that MCP-1 is an important mediator in several neuroinflammatory and neurodegenerative brain diseases characterized by neuronal degeneration such as PD.

MCP-1 levels in the blood are heightened in PD subjects compared to controls and correlate with PD progression [83].

Furthermore, it has been shown that MCP-1 could be implicated not only in disease progression but also in pathogenesis. The Ccl2-2518A allele is associated with lower MCP-1 production and reduced transcriptional activity following IL-1*β* stimulation [84], and in genetic epidemiological studies, possession of this allele is associated with a delayed onset of PD compared with patients expressing the Ccl2-2518G allele [85].

### 2.5. TNF-*α*

TNF-*α* is a proinflammatory cytokine well known for its role in chronic peripheral and central inflammation. TNF-*α* functions are mediated by two receptors: TNF-R1 (TNF-RSF1a) and TNF-R2 (TNFRSF1b). TNF-R1 is expressed in most tissues while TNF-R2 is found in limited cell types including cells of the immune system, oligodendrocytes, and certain neuron subtypes [10]. Both types of receptors are also expressed in the cortex, the subventricular zone of the lateral ventricle, and the hippocampus [86]. In homeostatic conditions, the TNF-*α* gene expression is low but increases dramatically in stressing conditions such as infection, trauma, and pathologies. In the CNS, TNF-*α* regulates a wide range of cellular processes and exhibits pleiotropic effects with positive or negative outcomes on the brain depending on concentrations and physiological or pathological state [87, 88]. Among the positive effects of TNF-*α*, there are increased neurogenesis and synaptic transmission [10, 89]. It has also been shown to be protective of hippocampal neurons by suppressing the accumulation of ROS and maintaining intracellular calcium levels [90]. Moreover, it modulates glutamatergic transmission, supports neural progenitor cell survival by mediating antiapoptotic signals via TNF-R2, and has a role in cognitive impairment, confirmed by investigations in TNF-*α* knock-out mice that showed reduced learning capabilities, than wild-type mice [91–93]. However, as reported in numerous other studies, TNF-*α* also has a dark face. It is notably involved in myelin damages [94], in favouring glutamate excitotoxicity [95], in the inhibition of long-term potentiation in the Cornu Ammonis area 1 (CA1) and dentate gyrus of the rat hippocampus, and in decreasing neurogenesis [96–98]. Furthermore, elevated levels of TNF have been described in many neurodegenerative situations such as in Alzheimer's disease (AD), multiple sclerosis (MS), amyotrophic lateral sclerosis (ALS), and PD [99–104].

High levels of TNF-*α* are found in both CSF and postmortem brain of PD patients and in animal models of PD [104–109] which may indicate that this cytokine acts as a mediator of neuronal damage. To understand the role of TNF-*α* in the neurodegenerative process, genetically modified mouse models were designed, such as knock-out mice lacking TNF-*α* or TNFR.

Knock-out mice for the TNF-*α* gene showed a decrease in dopamine content loss in the striatum after administration of 1-methyl-4-phenyl-1,2,3,6-tetrahydropyridine (MPTP) toxin and no difference in TH-positive cells in the nervous system suggesting a generally detrimental effect of TNF-*α* on the metabolism of dopamine [110] which is TNFR-independent [111].

However, TNF-*α* could also play a dual role in PD: neuroprotective during the early stages of the injury and neurotoxic during the chronic phase. In fact, Gemma and colleagues found that if TNF-*α* was inhibited early, i.e., within one week after administration of 6-OHDA, the inhibition could be neurotoxic; if TNF-*α* was inhibited late, i.e., 7 to 15 days after administration of 6-OHDA, the inhibition was neuroprotective [112].

Several *in vivo* reports [113–116] show detrimental effects of TNF-*α* injection or overexpression on the SN, but adverse results have also been reported, depending on the type, dosage, and administration regimen of TNF-*α*. Acute administration of TNF-*α* in the SN did not induce degenerative effect [113]. In contrast, in another study in which a much higher dose was administered, loss of dopaminergic cells in the SN at 14 days post inoculation was observed [114].

In experiments where TNF-*α* is expressed chronically, toxic effects of TNF-*α* were clearly observed. For instance, rats in which this cytokine was chronically expressed by intranigral injection of an adenoviral vector encoding TNF-*α* had, 14 days after adenoviral inoculation, akinesia of the forelimbs and a distinct inflammatory response in the brain [115]. The subsequent study by Chertoff and coworkers confirms the discovery discussed above; in this experiment, the chronic expression of TNF-*α* resulted in a progressive loss of dopaminergic (DA) neurons and their terminals in the nervous system and the recruitment of monocytes/macrophages [116].

Taken together, these results indicate that long-term expression of proinflammatory levels of TNF-*α*, or acute but very high expression of this cytokine, appears to be necessary to induce toxic effects on the SN while lower levels have been generating neuroprotection transient against 6-OHDA toxicity in the SN and striatum [116].

## 3. Physical Exercise in the Rehabilitation of Parkinsonian Subjects and Its Role in Neuroplasticity

Specific rehabilitation programs, as a support to pharmacological therapies in the treatment of parkinsonian patients, were proposed in 1956 [117]. However, in the beginning, the approaches were based only on empirical experience and there was no attempt to understand the underlying neurological mechanisms. In recent years, the benefits of exercise have been found to be linked to neuroplasticity [18]. To investigate the mechanisms by which exercise induces neuroplasticity in the mammalian brain, the loss of dopamine cells is induced by targeted injections of MPTP (mouse and nonhuman primate) or 6-OHDA in rats. In both models [118–123], physical exercise improves motor performance, including gait speed, step length, and balance.

Studies on the neuroprotective effects of physical exercise introduced forced or voluntary exercise before, during, or immediately after administration of the toxins (6-OHDA or MPTP) and reported improved motor functions, along with the preservation of dopaminergic neurons and the restoration of dopaminergic terminals in the striatum. These improvements have been mainly attributed to either an increased level of neurotrophic factors such as BDNF or GDNF [124–126] or exercise-induced downregulation of the dopamine transporter (DAT) [119, 123]. Other factors affect/modulate the neuroprotective effects of exercise, among which the temporal interval between the lesion and the beginning of the physical training (e.g., exercise started 1 week after toxin administration fails to protect against cell loss [127]) and the extent of toxin-induced damage.

Neurorestoration is suggested as another exercise-induced process for recovery of behavioural functions, and it does not involve neuroprotection [123]. In fact, neurorestorative effects of exercise are defined as the brain's responses to exercise after the completion of toxin-induced cell death. Studies have shown that exercise increases dopamine release, increases synaptic plasticity, and decreases dopamine clearance by reducing DAT expression [119, 128, 129]. Furthermore, it has been shown that strenuous exercise, on a treadmill, reverses the reduction of dopamine D2 receptors in the dorsal striatum, which usually occurs after injury [118]. Both the restoration of dopamine D2 receptors and the increase in dopamine release are extremely important in the advanced phase of motor learning when automaticity develops [130]. Therefore, both phenomena could contribute to the neuroplastic mechanisms involved in the improvement of exercise-induced motor behaviour and restoration of automaticity.

Physical exercise also modulates glutamatergic neurotransmission. Among the crucial aspects underlying motor impairment in individuals with PD, there is the hyperexcitability in the indirect pathway induced by dopamine depletion in the striatum in response to alterations in glutamate receptor expression and neurotransmitter release [131]. VanLeeuwen and colleagues have shown that strenuous exercise can restore the expression of glutamate receptors, including the *α*-amino-3-hydroxy-5-methyl-4-isoxazolepropionic acid (AMPA) receptors, which are modified in many neurological disease states and are considered a viable target for drug treatment [132, 133]. In addition to the effects on glutamate receptors, exercise can also alter the storage and release of glutamate in presynaptic terminals, which may also improve circuit function and reduce the increased inhibitory drive of the dopamine-depleted striatum [134–136]. Thus, these findings suggest that exercise, through its effects on neurotransmitters and their receptors, could help restore the neurophysiological properties of synapses within the damaged striatum that are necessary for normal motor learning and motor activity [18].

In summary, exercise is generally accepted as an intervention that could help both motor and nonmotor complications of PD, but it should be emphasized that not all types of rehabilitation approaches could facilitate neuroplasticity and behaviour in individuals with PD. Indeed, experience-dependent neuroplasticity is largely dependent on the intensity, repetition, specificity, difficulty, and complexity of the practice, and it is very likely that patients with PD need more time to achieve effective learning and automation. A precedent study by Frazzitta and colleagues [137] demonstrated that the rate of recurrence of physiotherapy sessions (2 daily sessions, 5 days a week for 4 weeks) induces beneficial effects that persist for a follow-up period of 12 months, with a reduced need to increase the doses of Levodopa. This result would suggest that the frequency of rehabilitation intervention is a critical factor, which could influence the natural progression of motor impairment in PD.

The study of Tinazzi and colleagues (2019) based on a four-week trunk-specific exercise program in PD patients with pronounced forward trunk flexion has confirmed the importance of intensive and specific physiotherapy. Rehabilitative protocols lasted 4 weeks (60 min/day, 5 days/week) and have led to improved passive and active control of the trunk that was maintained at one month post treatment [138].

Similarly, Corcos and colleagues [139] reported that progressive resistance exercise improved motor subscale Unified Parkinson's Disease Rating Scale (UPDRS-III) scores in PD patients with an effect lasting up to 2 years. Therefore, it could be hypothesized that the association of periodic intensive rehabilitation courses with pharmacological treatment should be considered one of the best options for the treatment of PD patients. To date, however, there is still a need for a general consensus on which is the best treatment modality (type-frequency-intensity) and on the most significant outcome measures [140].

### 3.1. Effects of Exercise on Cytokines and Neurotrophin Levels

Here, we intend to focus on the effects of exercise on altered levels of proinflammatory cytokines and GDNF and BDNF.

Neurotrophins are a group of proteins having the ability to stimulate survival, cell growth, and maintenance of the functional capacities of specific neuronal populations [141]. Initially, neurotrophins are synthesized as precursor proteins (proneurotrophins) and, because of the involvement of several enzymes, are converted into their mature form and released into the extracellular space [142]. Each of these mature proteins forms a complex with a twin molecule forming a dimeric structure that allows the activation of specific receptors [143]. Neurotrophins act through two types of receptors: tyrosine kinase receptors, with high affinity for mature neurotrophins, and p75 receptors, with low affinity for mature and high affinity for immature forms. Previous studies have suggested that proneurotrophins, through the p75 receptors, exert opposite biological effects with respect to mature proteins, and therefore, the proteolytic cleavage of proneurotrophins may represent a control mechanism that orchestrates the activity of neurotrophins [144]. Furthermore, these proteins are able to self-regulate their production as well as regulate the production of other members of this group of proteins [145, 146].

The most studied neurotrophic factors in PD are GDNF and BDNF.

GDNF is a neurotrophic factor purified for the first time from a rat glioma cell line (B49) [147] and belongs together with neurturin (NRTN), artemin (ARTN), and persephin (PSPN) to the family of GDNF ligands (GFL) belonging in turn to the superfamily of transforming growth factor *β* (TGF-*β*) [148]. In recent years, both the GDNF and the GFL ligands have been investigated due to their involvement in the survival of dopaminergic and noradrenergic neurons. However, GDNF besides acting on dopaminergic neurons promotes the survival of many other neuronal populations including motor and enteric neurons, noradrenergic and serotonergic cell population, and peripheral sensory and autonomic neurons. In addition, GDNF is expressed in brain regions that receive catecholaminergic afferents [149], such as the striatum and thalamus [150, 151].

Furthermore, studies performed on rat and mouse models of PD showed the neurorestorative properties of GDNF [152, 153]. In nonhuman primate PD models, GDNF augmented the sizes of nigral DA neurons that were 20% larger, with an increased fiber density, and it improved parkinsonian symptoms such as bradykinesia, stiffness, balance, and posture [154, 155].

Furthermore, the trophic effects of GDNF have been described as TGF-*β*-dependent. Indeed, TGF-*β* acts as a modulator of GDNF signalling and participates in the translocation of GDNF family receptor-*α* (GFR*α*) coreceptors in the cell membrane. The association between ligand and coreceptor forms the GDNF-GFR*α* complex that can interact with the neural cell adhesion molecule (NCAM) receptors or with a transmembrane tyrosine kinase (RET (REarranged during Transfection)) dimer, inducing their homodimerization and tyrosine autophosphorylation and initiating the intracellular signalling process. Hence, a series of cascades occur, including the activation of the nonreceptor tyrosine kinase Fyn- (Fyn-) focal adhesion kinase- (FAK-) MAPK signalling pathway by the GDNF-GFR*α*-NCAM complex and the activation of the rat sarcoma virus GTP-binding protein- (RAS-) MAPK-phosphoinositide 3-kinase (PI3K) signalling pathway by the GDNF-GFR*α*-RET complex [156, 157]. These cascades play a role in the control of neurite outgrowth [158] and in neuronal growth and survival through the activation of the cAMP response element-binding protein (CREB) and the protein kinase B (PKB), involved in cell proliferation and transcription [156, 159]. Furthermore, GDNF also appears to be able to modulate microglia activation through GDNF family receptor *α*1 (GFR*α*1). Thus, GDNF triggers signalling cascades, which are responsible for inhibiting microglia activation [160].

Because of these promising effects on PD, researchers have investigated several means able to increase GDNF levels.

The direct delivery of GDNF to the brain region affected in PD seems to optimize the chances of obtaining therapeutic efficacy. Using different viral vectors and different animal models including adeno-associated viral vectors (AAV) in rat models of PD [161], AAV in nonhuman primates [162], and lentivirus [163] and adenovirus [164] in rats, the neurorestorative effects of GDNF were carefully demonstrated. Although these findings are promising, the results from clinical trials are not very encouraging. For example, a study based on monthly intracerebroventricular injections of GDNF reported no improvement and several side effects [165]. However, another study where GDNF was administered directly into the putamen showed an improvement in motor function as well as an increase in dopamine uptake measured by positron emission tomography (PET) without any side effects [166].

So far, the clinical evaluations of GDNF treatments in patients with PD have been inconsistent, potentially due to insufficient distribution of GDNF throughout the nigrostriatal system [167–169]. In order to increase GDNF nigrostriatal distribution, we conducted a study using an implantable and removable encapsulated cell system able to deliver targeted and long-lasting *de novo* synthesized high levels of human GDNF into the striatum of 6-OHDA-lesioned rats and Goettingen miniature pig. GDNF was distributed throughout the striatum, and this massive spreading of the protein led to almost complete protection of dopaminergic neurons in the damaged SN and preservation of TH-positive fibers in the striatum. Furthermore, these same animals demonstrated a slow and steady improvement in motor performance when evaluated on 3 separate neurological tests (cylinder, placing, and stepping tests). Our data demonstrated also that a part of the motor recovery is explained by the germination or regeneration of residual dopaminergic terminals postinjury [170]. Beneficial effects were observed when the same therapeutic approach was investigated into the hippocampus of pilocarpine-treated rats [171, 172].

Thus, long-term targeted release of GDNF over the majority of the nigrostriatal system could represent an interesting and attractive option for treatment of PD.

Another valuable ally for increasing GDNF release is physical exercise [125]. A very recent study also highlighted the ability of controlled exercise on a treadmill in mice to increase the striatal content of GDNF as well as normalize striatal levels of tyrosine hydroxylase and attenuate L-DOPA-induced dyskinesia (LID [173]), thus providing the first indication that the antidyskinetic effects of exercise may lead to an increase in striatal GDNF levels [174].

The other most studied neurotrophic factor in PD is BDNF. BDNF supports the survival and the differentiation of dopaminergic neurons and protects them from neurotoxin-induced degeneration [175]. Many studies have documented some evidence of a decreased expression of BDNF in different neurodegenerative diseases [176, 177]. PD patients present lower concentrations of BDNF mRNA and protein in the substantia nigra pars compacta than healthy controls [178, 179]. On the contrary, some studies reported an increase of BDNF levels in the serum of PD patients, especially in moderate to severe stages of the disease [180, 181]. This could happen because the CNS to counteract neuronal loss would increase BDNF production resulting in enhanced serum levels of the protein. However, there is no direct evidence that supports this hypothesis. The onset and progression of PD are also associated with neuroinflammation. Sawada and coworkers have found a notable increase of microglial cells in the hippocampus, amygdala, and entorhinal cortex of PD patients, which was associated with a decrease of BDNF mRNA expression and increased IL-6 in those regions. Moreover, they have also shown increased levels of IL-1*β*, interleukin-2 (IL-2), IL-6, and TNF-*α* in the striatum of PD patients associated with decreased BDNF protein levels in the same structure [182]. However, there is no evidence on how changes in BDNF levels in the brain affect the progression of PD, and further analysis of the interaction between proinflammatory cytokines and BDNF levels is necessary.

A research field in continuous development focuses on studying the effects of exercise on BDNF level changes in healthy adult populations [183, 184] and in people affected by neurodegenerative disease [185, 186].

Exercise-induced BDNF release seems to carry out a crucial role in neuroplastic effects of rehabilitation interventions in humans with neurodegenerative disease, particularly with PD [183, 187–189], and it is believed that the physiologic mechanisms underlying exercise-induced BDNF changes in PD could include long-term potentiation (LTP) and long-term depression (LTD) mechanisms [190, 191].

In fact, it seems that BDNF plays a complicated role in both LTP and LTD and contributes in different ways to short-term and long-term plasticity: initially, the pro-BDNF binds to two postsynaptic receptors: the tyrosine kinase B (TrkB) receptor and the p75 receptor. TrkB activation facilitates the induction of LTP [192] while p75 receptor stimulation modulates the N-methyl-D-aspartate (NMDA) activity that promotes the subsequent induction of LTD [193]. Thus, although its action is particularly complex, BDNF is a major player in synaptic plasticity.

In order to explore if the neuroprotection offered by exercise is BDNF-dependent, Gerecke and colleagues (2010) studied the effectiveness of voluntary physical training with a running wheel in mice on a 90-day program. Mice were divided into two groups: mice with heterozygous deletion of the BDNF gene and wild-type mice. Only the second group showed neuroprotection against exposure to the toxin inducing dopamine cell loss [194]. Researchers also analysed voluntary training in PD mice after periods of 30, 60, or 90 days. The running training for 90 days best promoted a neuroprotective effect on dopaminergic cells showing only a 9% loss of DA neurons while loss of DA neurons was more consistent in animals that underwent 30 days or 60 days of voluntary training [194].

A different research team has demonstrated that physical exercise reduces the 6-OHDA-induced damage acting on BDNF receptors. In fact, blocking of BDNF receptors causes enhanced postlesion nigrostriatal dopaminergic cell loss, quantified as a reduction in the expression of TH [126, 191].

Finally, clinical data on the impact of physical exercise on reducing PD-related proinflammatory cytokine levels received increasing attention over recent years; in particular, investigations focus on the modulation of inflammatory markers as potential molecular mechanisms involved in the beneficial effects of exercise on PD patients.

Cadet and colleagues showed that cyclical exercise, performed for months, leads to a significant increase in the plasma level of anti-inflammatory signal molecules, such as interleukin-10 (IL-10) and adrenocorticotropin, while plasma levels of proinflammatory cytokines such as IL-1 and IL-6 were not affected. Additionally, this cyclic exercise protocol has also been shown to improve fine motor skills. These data suggest that cyclical exercise induces the formation of anti-inflammatory signalling molecules, which appear to be associated with relieving of some clinical impairments of PD [195].

Two more recent studies (years 2017 and 2018) also showed that alternative and not traditional physical exercises such as Qigong, an oriental mind-body exercise, or physical exercise in water can improve the inflammatory state of PD.

In this study by Moon and colleagues, ten subjects with PD were recruited and then randomly assigned to one of the two groups who received six weeks of Qigong intervention (experimental group) or sham Qigong (control group). After the intervention, the serum level of TNF-*α* in the experimental group was significantly reduced in all subjects, and there is a stabilized sleep pattern suggesting that TNF-*α* can potentially affect sleep quality in people with PD [196].

Pochmann and colleagues instead focused on exploring the molecular mechanisms underlying the improvement of motor symptoms and functional mobility in water-based exercise interventions in patients with PD. The authors reported higher levels of the proinflammatory cytokines IL-1*β* and MCP-1 in patients with Parkinson's compared to the control group and a reduction in the levels of these proinflammatory cytokines after an aquatic physiotherapy program for 1 month, two times a week (60 min/session). These data support the idea that the inflammatory state is linked to PD and that proinflammatory cytokines could be considered promising biomarkers for the diagnosis and progression of this condition [197].

## 4. Conclusion

In conclusion, both traditional and not traditional forms of exercise have been shown to be important for improving motor function, facilitating neuroplasticity, and reducing neuroinflammation in PD. Further investigations are needed to broaden our knowledge on the mechanisms through which specific physical training induces neuroplasticity, eventually leading to a deeper knowledge of its role in interfering with the disease progression and to identify novel therapeutic targets to finally improve the effects of pharmacological approaches of PD.

## Abbreviations

AD: Alzheimer's disease
ALS: Amyotrophic lateral sclerosis
AMPA receptors: *α*-Amino-3-hydroxy-5-methyl-4-isoxazolepropionic acid receptors
ARTN: Artemin
AVV: Adeno-associated viral vectors
BBB: Blood-brain barrier
BDNF: Brain-derived neurotrophic factor
BMECs: Brain microvascular endothelial cells
CA1: Cornu Ammonis area 1
CNS: Central nervous system
CREB: cAMP response element-binding protein
CSF: Cerebrospinal fluid
DA neurons: Dopaminergic neurons
DAT: Dopamine transporter
ERK: Extracellular signal-regulated kinase
FAK: Focal adhesion kinase
Fyn: Nonreceptor tyrosine kinase Fyn
GDNF: Glial cell line-derived neurotrophic factor
GFR*α*: GDNF family receptor-*α*
GFR*α*1: GDNF family receptor *α*1
GFL: GDNF family
JAK/STAT signalling pathway: Janus kinase- (JAK-) signal transducer and activator of transcription (STAT)
IFN-*γ*: Interferon-*γ*
IL-1*β*: Interleukin-1*β*
IL-2: Interleukin-2
IL-6: Interleukin-6
IL-8: Interleukin-8
IL-10: Interleukin-10
LID: L-DOPA-induced dyskinesia
LPS: Lipopolysaccharide
LTD: Long-term depression
LTP: Long-term potentiation
MAPK: Mitogen-activated protein kinase
MCP-1: Monocyte chemotactic protein-1
MPTP: 1-Methyl-4-phenyl-1,2,3,6-tetrahydropyridine
MS: Multiple sclerosis
NCAM receptors: Neural cell adhesion molecule receptors
NMDA: N-Methyl-D-aspartate receptor
NRTN: Neurturin
6-OHDA: 6-Hydroxy dopamine
PI3K: Phosphoinositide 3-kinase
RNS: Reactive nitrogen species
ROS: Reactive oxygen species
SN: Substantia nigra
PD: Parkinson's disease
PET: Positron emission tomography
PKB: Protein kinase B
PSPN: Persephin
RAS: Rat sarcoma virus GTP-binding protein
RET: REarranged during Transfection
TGF-*β*: Transforming growth factor *β*
TH: Tyrosine hydroxylase
TNF-*α*: Tumor necrosis factor-*α*
TrkB: Tyrosine kinase B
UPDRS-III score: Unified Parkinson's Disease Rating Scale score.
